# Lipidome atlas of p53 mutant variants in pancreatic cancer

**DOI:** 10.1186/s13062-025-00635-w

**Published:** 2025-04-11

**Authors:** Kian Cotton, Charley Comer, Sabrina Caporali, Alessio Butera, Stephanie Gurres, Francesco Capradossi, Angelo D’Alessandro, Ivano Amelio, Maria Victoria Niklison-Chirou

**Affiliations:** 1 Life Science Department, University of Bath, Claverton Down, Bath, BA2 7AY UK; 2 Chair for Systems Toxicology, University of Konstanz, Constance, Germany; 3 University of Colorado Anschutz Medical Campus, Aurora, CO 80045 USA; 4 Blizard Institute, Barts and The London School of Medicine and Dentistry, Queen Mary University of London, London, UK

**Keywords:** p53 mutant, Lipid, Cancer, Metabolism

## Abstract

Mutations in the tumour suppressor protein p53 are present in 70% of human pancreatic ductal adenocarcinomas (PDAC), subsequently to highly common activation mutation of the oncogene KRAS. These p53 mutations generate stable expression of mutant proteins, such as p53^R175H^ and p53^R273H^, which do not retain p53 wild type function. In this study, we investigated the impact of two specific p53 mutant variants on lipid metabolism of pancreatic cancer. Lipids critically participate to tumorigenesis with to their roles in membrane biosynthesis, energy storage and production of signalling molecules. Using cell lines derived from mouse models of PDAC generated by knock-in p53 alleles carrying point mutations at codons R172H and R270H (equivalent to R175H and R273H in humans), we found that silencing p53^R172H^ and p53^R270H^ in pancreatic cancer cells significantly alters lipid metabolism, with patterns of common and variant specific changes. Specifically, loss of p53^R172H^ in these cells reduces lipid storage. Additionally, silencing either p53^R172H^ or p53^R270H^ individually leads to marked increases in lysophospholipid levels. These findings offer new insights into the lipidome reprogramming induced by the loss of mutant p53 and underscore changes in lipid storage as a potential key molecular mechanism in PDAC pathogenesis.

## Introduction

With a dismal 5-year survival rate of under 5%, pancreatic ductal adenocarcinoma (PDAC) ranks as the 5th most common cause of cancer-related mortality in both Europe and the United States [1, 2]. This prognosis is largely attributed to the disease's aggressive progression, as nearly 90% of individuals are diagnosed when the cancer is already in an advanced, non-resectable state and demonstrates significant resistance to chemotherapy [3]. PDAC originates from precursor lesions termed pancreatic intraepithelial neoplasms (PanINs), which develop through a stepwise accumulation of genetic alterations. These typically involve mutations in the KRAS oncogene and the inactivation of tumor suppressor genes such as CDKN2A, TP53, and SMAD4 [4, 5]. Although these genetic changes are well-documented in PDAC, their precise contributions to cancer initiation and progression remain incompletely understood.

Mutations in the *TP53* gene, encoding the tumour suppressor protein p53, are found in approximately 70% of PDAC cases, often occurring subsequent to activating mutations in the *KRAS* gene [6–9]. A major consequence of p53 inactivation is the loss of capacity of cell cycle regulation and cell death, associated to onset of genomic instability [10, 11]. Unlike many tumour suppressor genes that are inactivated by deletions or nonsense mutations, TP53 mutations typically result in the expression of stable mutant proteins rather than the complete loss of p53 protein levels [12, 13]. These mutant p53 proteins, such as p53^R175H^ and p53^R273H^, not only lose their tumour-suppressing functions but can also acquire new oncogenic properties that drive cancer progression [14, 15], although the significance of these gain-of-function effects remain controversial [16–18].

Recent studies have highlighted the importance of lipid metabolism in several cancers, including PDAC [19]. Lipids are essential for several key cellular processes; neutral lipids, for instance, serve as energy reserves that can be metabolised through β-oxidation to meet the high energy demands of rapidly proliferating cancer cells [20]. Furthermore, phospholipids, which constitute the primary components of cell membranes, are critical for maintaining membrane integrity and facilitating cancer cell migration and invasion [21]. Lysophospholipids act as bioactive signalling molecules, playing a role in cell communication, proliferation, and survival [22].

Aberrant lipid metabolism is a newly accepted hallmark of cancer and has been implicated in key aggressive characteristics such as survival, growth, and metastasis [23–25]. In PDAC, alterations in lipid metabolism can support the biosynthesis of new membranes required for cell division, provide energy through fatty acid oxidation, and generate signalling molecules that promote required oncogenic pathways [26]. Despite the recognised importance of lipid metabolism in cancer, the specific impact of mutant p53 on lipid metabolism in PDAC has received limited research.

In this study, we employed cell lines derived from a genetically engineered mouse model of pancreatic cancer with knock-in p53 alleles harbouring point mutations at codons R172H and R270H (equivalent to R175H and R273H in humans) to investigate the effects of mutant p53 on lipid metabolism. We revealed that silencing these mutant p53 proteins in pancreatic cancer cells leads to significant alterations in lipid storage and composition, particularly in lysophospholipid levels. Specifically, the loss of p53^R172H^ reduces lipid storage, while silencing either p53^R172H^ or p53^R270H^ individually results in marked increases in lysophospholipid levels.

Our study provides an atlas of the mutant p53-induced lipid landscape in PDAC, suggesting changes in lipid storage and signalling may be critical molecular mechanisms in PDAC pathogenesis.

## Results

### p53 mutant-dependent lipidome of pancreatic cancer cells

**Fig. 1:**
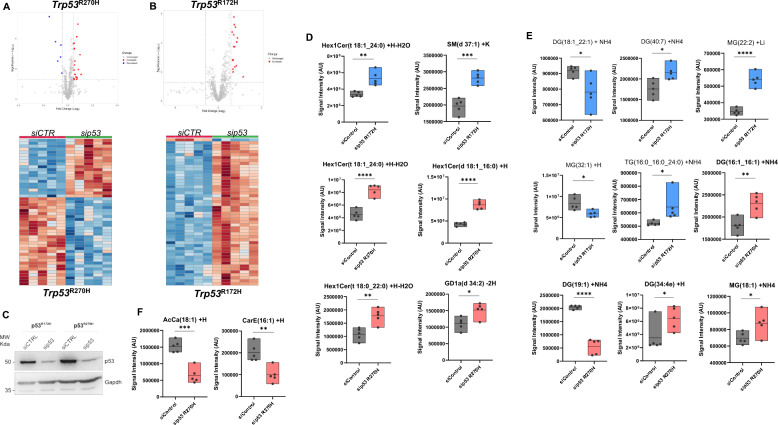
p53 silencing remodels the lipidome of pancreatic cancer cells. **A**, **B** Volcano plots and heat-maps were obtained from all identified lipid compounds in KPC cells expressing p53^R172H^ or p53^R270H^ following transfection with siRNA Control or siRNA against p53. Heat maps of endogenous metabolites between control groups and sip53 (n = 5). In the heat map, red-filled and bleu-filled lines indicate increased or decreased levels of lipids, respectively. **C** Western blot analysis reports silencing efficiency of p53 mutants in KPC cells following transfection of siRNA Control or siRNA against p53. **D–F** Box plots show the significantly modulated lipid entities after p53 mutant knockdown. *p value < 0.05; **p value < 0.01; ***p value < 0.001; ****p value < 0.0001. *p* value was calculated by unpaired two-sample t-test

The two most common mutations in the p53 protein in human pancreatic cancer, R175H and R273H (equivalent to R172H and R270H in mouse), result in the expression of a stable mutant protein [27, 28]. To test the regulatory role of these mutant p53 forms on the pancreatic cancer lipid profile, we carried out a global untargeted lipidomic profiling in KPC cells derived from mouse PDAC, generated by the pancreas-specific expression of the constitutively active KRAS^G12D^ (pdx1-CRE LSL-KRAS^G12D^) and mutant-p53 R172H or R270H expression. Here, we depleted KPC cells from the respective p53 mutant with an siRNA-mediated silencing. 884 different lipid compounds were identified and compared upon p53 mutant silencing with their scramble transfected respective control condition (Fig. 1A–C). This initial analysis indicated that depletion of mutant-p53 extensively remodels the lipidome of pancreatic cancer cells.

We next grouped lipids in 33 individual classes to produce an overview of those groups with differential expression following depletion of each mutation (Table 1). Sphingolipids play an essential structural role in cell membranes, as well as being potent signalling molecules [29]. An increase in Hex1Cer and SM were observed in p53^R172H^ depleted cells, and a similar trend was observed for Hex1Cer levels upon silencing of R270H (Fig. 1D). A significant increase in sphingolipid levels, such as hexosylceramides (Hex1Cer), sphingomyelin (SM), and disialoganglioside (GD1a), was observed after silencing of mutant p53^R270H^, when compared with control groups (Fig. 1D).

**Table 1:** Summary signals for the identified lipid classes between sip53 and control groups

Class	KPCR172H siControl	KPCR172H sip53	t-test	KPCR270H siControl	KPCR270H sip53	t-test
AcCa	2.60E+06	2.84E+06	0.20139	2.74E+06	2.34E+06	0.20186
AcylCoA	3.55E+05	**4.36E+05**	0.04952	4.39E+05	*3.57E+05*	0.05155
AEA	4.18E+05	4.05E+05	0.51956	2.12E+05	1.92E+05	0.59221
CarE	3.42E+05	4.46E+05	0.43697	2.03E+05	*9.01E+04*	0.00969
Cer	5.87E+06	*5.30E+06*	0.00059	5.66E+06	**7.37E+06**	0.00004
ChE	8.15E+05	9.14E+05	0.70490	8.73E+05	*6.41E+05*	0.30994
CL	1.35E+07	1.40E+07	0.30651	1.32E+07	1.76E+07	0.01083
Co	5.91E+05	5.27E+05	0.32791	3.77E+05	**5.91E+05**	0.07582
DG	6.79E+06	*5.99E+06*	0.00640	9.17E+06	**1.04E+07**	0.00416
DLCL	1.58E+06	**2.06E+06**	0.00377	2.39E+06	2.74E+06	0.74871
FA	2.89E+08	2.74E+08	0.26061	2.77E+08	2.78E+08	0.28493
GD1a	1.92E+06	2.01E+06	0.31994	3.67E+06	**5.51E+06**	0.07963
GM3	5.14E+05	5.56E+05	0.92182	9.88E+04	**2.30E+05**	0.03891
GT3	1.48E+06	1.58E+06	0.59084	2.17E+06	**3.50E+06**	0.03091
Hex1Cer	2.41E+06	**3.57E+06**	0.03370	3.40E+06	**7.04E+06**	0.01095
LPC	3.98E+06	4.30E+06	0.18253	3.11E+06	*2.79E+06*	0.00264
LPE	8.90E+05	**1.09E+06**	0.01563	2.60E+06	2.76E+06	0.09759
LPG	4.48E+05	**6.90E+05**	0.09905	1.05E+06	1.28E+06	0.12826
LPI	1.94E+06	**2.28E+06**	0.00188	5.31E+06	5.91E+06	0.06143
LPS	1.68E+06	1.36E+06	0.33132	1.94E+06	1.81E+06	0.58922
MG	3.39E+06	*2.75E+06*	0.07715	1.03E+07	1.03E+07	0.87416
MLCL	2.62E+06	2.65E+06	0.85808	4.32E+06	4.54E+06	0.31699
OAHFA	3.98E+06	3.91E+06	0.70963	4.22E+06	*3.90E+06*	0.04667
PA	1.31E+06	*1.09E+06*	0.00418	1.66E+06	**2.33E+06**	0.00100
PC	3.78E+07	3.88E+07	0.16859	3.22E+07	3.55E+07	0.00003
PE	3.08E+07	3.31E+07	0.03162	2.65E+07	**3.26E+07**	0.00392
PG	9.62E+06	*6.46E+06*	0.29773	7.67E+06	**9.27E+06**	0.09447
PI	7.57E+06	7.12E+06	0.05216	8.70E+06	**9.72E+06**	0.00177
PS	1.82E+07	1.73E+07	0.10862	1.62E+07	**2.14E+07**	0.02495
SM	2.42E+07	*2.09E+07*	0.02189	3.08E+07	3.07E+07	0.86963
SPH	2.83E+06	3.02E+06	0.19443	1.37E+06	1.18E+06	0.55418
ST	1.12E+06	1.16E+06	0.91563	2.97E+06	3.20E+06	0.16045
TG	1.36E+06	1.42E+06	0.02054	1.54E+06	1.52E+06	0.29237

The identified lipids influenced by silencing of mutant p53 (172H and 270H) were grouped into four lipid categories: neutral lipids, sphingolipids, phospholipids and fatty acyl and other lipids. The 33 lipid classes were as follows: **phospholipids:** lysophosphatidylcholine (LPC), lysophosphatidylethanolamine (LPE), phosphatidylserine (PS), phosphatidylinositol (PI), phosphatidylglycerol (PG), Phosphatidic acid (PA), phosphatidylethanolamine (PE), phosphatidylcholine (PC), lysophosphatidylinositol (LPI), monolysocardiolipin (MLCL), dilysocardiolipins (DLCL) and cardiolipin (CL); **sphingolipids**: sphingomyelin (SM), ceramides (Cer), sphingomyelin (SM), hexosylceramides (Hex1Cer), gangliosides (GM3), disialoganglioside (GD1a)*,* trisialosyl lactosylceramide (GT3) and sphingosine (SPH), **neutral lipids**: cholesterol ester (ChE), sterol (ST), monoglycerol (MG), diglyceride (DG), and triglyceride (TG); and **fatty acyl and other lipids**: N-acylethanolamine (AEA), O-Acyl-ω-hydroxy Fatty Acid (OAHFA), fatty acids (FA), lyso-phosphatidyl-glycerols (LPG), lyso-phosphatidylserine (LPS) and coenzyme (Co) and acyl carnitine (AcCa). The summary signals for the identified lipid classes between silencing of mutant p53 and control groups are presented in Table 1

Additionally, we looked into the effect of silencing mutant p53 on expression of neutral lipids such as diacylglycerols (DGs), triacylglycerols (TGs) and monoacylglycerols (MGs), which provide cancer cells with inert forms of energy used in conditions of nutrient deprivation [20]. Interestingly, we observed that silencing of R172H and R270H has significant effects on neutral lipids. A significant decrease in DG (18:1_22:1) and an increase in DG (40:7) levels were observed after silencing R172H but, conversely, an increase in DG levels, namely DG (16:1_16:1) and DG (34:4e), and a decrease in DG (19:1) were observed after silencing R270H. MG (22:2) and TG (16:0_16:0_24:0) were found to be upregulated whilst MG (32:1) was significantly downregulated after silencing of R172H. Also, MG (18:1) was significantly upregulated after silencing of p53^R270H^ (Fig. 1E). Thus, divergence in the role of the two p53 mutant variants emerged in the regulation of neutral lipids.

Furthermore, acylcarnitine (AcCa) and carboxylesterase (CarE) as constituents of fatty acyl compounds showed a dramatic reduction in silenced R270H cells (Fig. 1F). AcCa levels are essential for beta oxidation within cancer cells and thus these data indicate that different mutant p53 proteins can differentially impact the energy stores required for the rapid proliferation of pancreatic cancer cells.

### Mutant p53 regulates the production of lysophospholipidome

It was demonstrated that lysophospholipid species such as lysophosphatidylethanolamine (LPE), lysophosphatidylcholine (LPC) and lysophosphatidylserine (LPS) are influenced by wild-type p53 in pancreatic cancer [19]. The levels of such species have already been implicated in numerous pathophysiological conditions such as cancer, fibrosis, inflammation neurodegenerative diseases as well as autoimmune diseases [22, 30]. Therefore, we looked into lysophospholipids level upon mutant p53 manipulation in pancreatic cancer.

**Fig. 2:**
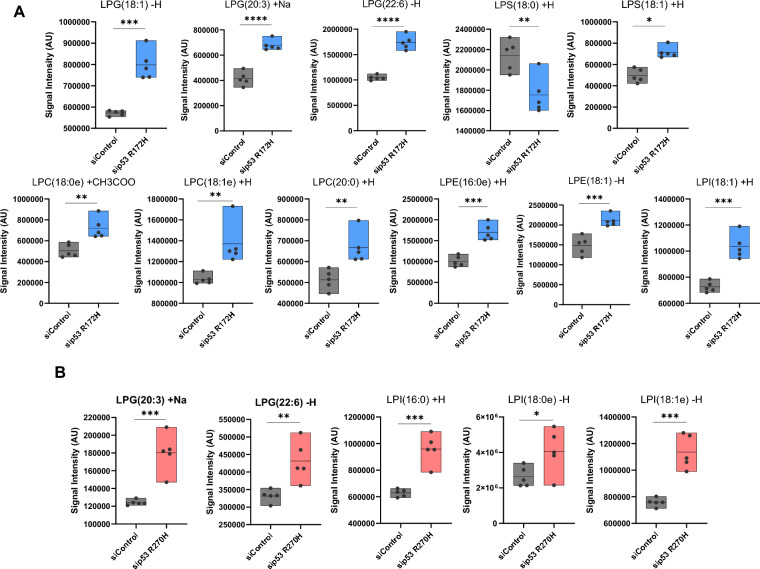
Lisophospholipids are affected by p53 mutant. **A** Box plots show the significantly modulated lipid entities after p53^R172H^ knockdown. *p value < 0.05; **p value < 0.01; ***p value < 0.001; ****p value < 0.0001. **B** Box plots show the significantly modulated lipid entities after p53^R270H^ knockdown. *p value < 0.05; **p value < 0.01; ***p value < 0.001; ****p value < 0.0001. *p*value was calculated by unpaired two-sample t-test

Our analysis, however, revealed also altered lysophospholipids abundance, with lysophosphatidylinositol (LPI), LPE, and lysophosphatidylglycerols (LPG) being the most affected lysophospholipids after silencing of p53 R172H (Fig. 2A). p53^R270H^ appears to mainly affect some species of LPI and LPG (Fig. 2B). LPC, the most abundant class of lysophospholipids in plasma, was notably increased also after silencing of R172H (Fig. 2A). Conversely, LPS (18:0) was decreased whilst LPS (18:1) was increased upon p53^R172H^ depletion, although the change in absolute LPS was relatively small (Fig. 2A).

**Fig. 3:**
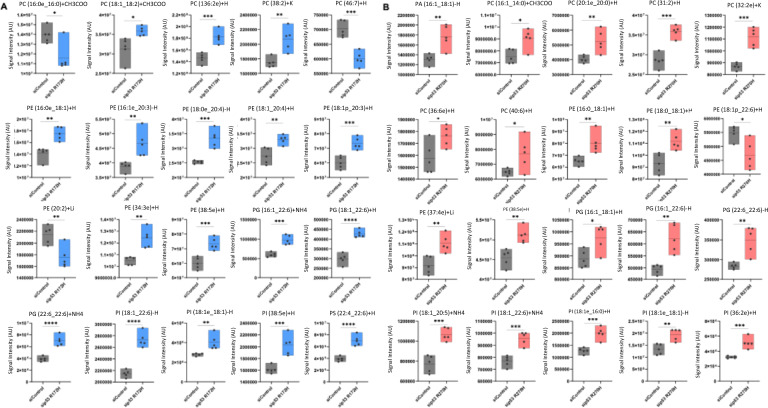
Other phospholipid species are altered by p53^R172H^ or p53^R270H^. **A**, **B** Box plots show the significantly modulated phospholipid content after p53^R172H^ (in **A**) or p53^R270H^ (in **B**) knockdown. *p value < 0.05; **p value < 0.01; ***p value < 0.001; ****p value < 0.0001. *p* value was calculated by unpaired two-sample t-test

Consistently, we observed an increase of phospholipid species such as, phosphatidylserine (PS), phosphatidylglycerol (PG), phosphatidylinositol (PI), phosphatidylethanolamine (PE), p, after silencing p53^R270H^. In contrast, p53^R172H^ depletion caused the reduction of PG levels but overall, the silencing of this specific mutant did not massively affected phospholipid species (Table 1). Phospholipids, which form the bilayer structures of all cellular membranes, have been shown to increase during periods of cell transformation and tumour progression [21]. Our analysis revealed a larger general cohort of intracellular PC, PE and PI species indicating that these species are the most affected by p53^R270H^ (Table 1). To gain deeper insights into the impact of mutant p53 on phospholipid composition, we conducted a comprehensive analysis comparing lipid species that exhibited differential abundance upon silencing of the R172H and R270H variants in pancreatic cancer cells (Fig. 3A, B). The most significantly altered phospholipids after the depletion of R172H were the PG(18:1), PG(22:6), PI(18:1) and PS(22_4). On the other hand, the most significantly altered phospholipids after loss of R270H were the PC(31:2), PC(32:2), PI(18:1) (Fig. 3A, B).

The formation of lysophospholipids is a consequence of the oxidative lipid damage and enzymatic activity associated with ferroptosis [31, 32]. During ferroptosis, the lipid peroxidation of polyunsaturatedfattyacids (PUFAs) in phospholipids generates lipid hydroperoxides [33]. This oxidative stress can lead to the cleavage of fatty acid chains from phospholipids, resulting in the formation of lysophospholipids. Ferroptosis can activate phospholipases, such as phospholipase A2 (PLA2), which hydrolyses the sn-2 acyl bond of phospholipids to produce lysophospholipids and free fatty acids [34]. This activity is part of the cellular response to oxidative damage and contributes to the breakdown of membrane integrity during ferroptosis. Hence, lysophospholipids abundancy can be linked to ongoing ferroptosis. Together, these results show that p53 gain of function mutations may drive aberrant phospholipid metabolism in pancreatic cancer cells.

### Mutant p53 regulates acyl-CoA levels in cancer cells

**Fig. 4:**
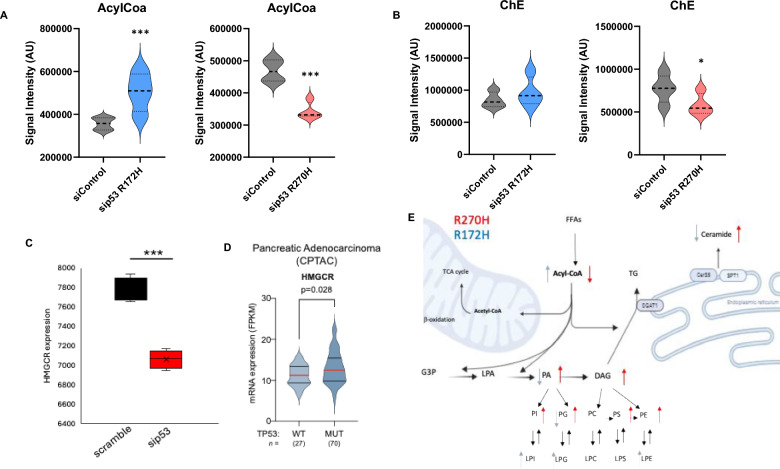
AcylCoA levels are significantly affected after loss of mutant p53. Violin plots showing the significantly modulated lipid classes after p53 knockdown. **A** AcylCoA species found significantly increased in sip53 (R172H) or sip53 (R270H) cells comparing with control groups.** B** Cholesterol ester (ChE) species found after sip53 (R172H) or sip53 (R270H) comparing with control groups. No significant changes in ChE levels were observed. **C** HMGCR mRNA level is influence by p53^R270H^ expression level. **D** HMGCR expression correlates with p53 mutant status in Pancreatica adenocarcinoma patients (CPTAC). **E** Summary of the lipid profiling changes after loss of mutant p53 in pancreatic cancer cells. Changes induced after sip53 (R172H) is shown in blue and after sip53 (R270H) are shown in red. Arrow up indicate increase and arrows down indicate reduction. *p value < 0.05; ***p value < 0.001. *p* value was calculated by unpaired two-sample t-test

Altered lipid metabolism, starting with acyl-CoA formation, is a key factor in the progression of various diseases, including cancer. For fatty acids to participate in metabolic pathways, they must first be converted into acyl-CoA. This activation enables their involvement in essential processes such as membrane phospholipid synthesis, energy storage, oxidation for energy production, and the generation of signaling lipids [35]. To explore the dysregulation of fatty acid metabolism in pancreatic cancer, we measured acyl-CoA levels after silencing the mutant p53 gene. We observed increased acyl-CoA levels following the silencing of the mutant p53 R172H variant (Table 1 and Fig. 4A). These data suggest a reduced use of the lipid pathway or increased incorporation of free fatty acids. Conversely, decreased acyl-CoA levels were observed after silencing the mutant R270H variant, indicating a higher demand for signalling lipids (Table 1 and Fig. 4B). Overall, these findings suggest that mutant p53 induces significant changes in lipid metabolism in pancreatic cancer cells and that these changes can vary depending on the specific p53 mutation.

### Cholesterol level is influenced by Mutant p53

Cholesterol is crucial for the survival and growth of mammalian cells [36]. It also modulates signalling pathways involved in tumorigenesis and metastasis by covalently modifying proteins, including those in the Hedgehog and Smoothened pathways, as well as the Rho signalling pathway [37]. Interestingly, we did not observe any significant change in cholesterol ester (ChE) levels after silencing the mutant p53 R172H variant, despite it being described as a common metabolic pathway regulated by p53 [36], potentially through increased uptake of cholesterol (Table 1, Fig. 4A). However, decreased ChE levels were observed after silencing the mutant R270H variant (Table 1, Fig. 4B). This finding is consistent with RNA sequencing data showing reduced levels of 3-hydroxy-3-methylglutaryl coenzyme-A reductase (HMGCR), the rate-limiting enzyme in the cholesterol synthesis pathway (Fig. 4C). Consistently with this regulation, expression level of HMGCR correlated with p53 mutational status in PDAC patients, as indicated by the analysis of cancer genomic dataset (CPTAC) (Fig. 4D). HMGCR was previously shown to be regulated and influenced as expression level by different p53 mutant forms in breast cancer [38, 39].

Overall, these results suggest that mutant p53 induces significant changes in cholesterol metabolism in pancreatic cancer cells and again highlights a clear divergence in metabolic profiles between the two mutations.

## Conclusion

Substantial progress has been made in the past years understanding the regulatory mechanisms of p53 in lipid metabolism. These metabolic processes, while supporting substrate availability for the rapid proliferation of tumour cells, might also present unique therapeutic opportunities. The role of mutant p53 in modulating the mevalonate pathway, particularly through enhanced activation of SREBP-2, already suggested a significant impact on isoprenoid and cholesterol biosynthesis [39], pioneering this area.

In this study, we present an atlas of lipid regulation influenced by two highly frequent p53 mutant variants in pancreatic cancer (Fig. 4E). Our analysis reveals both shared regulatory patterns and distinct variant-specific effects. This descriptive analysis underscores the necessity of a systematic assessment of p53 mutant variants to fully explore their potential as therapeutic targets and to determine the most effective strategies for targeting them. By mapping the lipidome regulation, this work aims to provide a comprehensive overview of the variant-specific effects of p53 mutants on lipid metabolism [12]. Our work aims to guide future research in this area, particularly studies that could uncover vulnerabilities in cancer and lead to viable therapeutic options. Such research would benefit from the careful selection of cancer patient cohorts harbouring p53 mutations.

## Materials and methods

### Cell culture

Pancreatic cancer cell lines from mouse models (KPC270 and KPC172) were cultured in DMEM (Gibco). Each medium was further supplemented with 10% (FBS, Gibco) and penicillin/streptomycin (2 units/ml) (Gibco), and the cell lines were maintained at 37 °C under 5% CO_2_.

### Transfection and gene silencing

siRNA transfection was carried out using Lipofectamine RNAiMAX (Invitrogen, Waltham, MA, USA). The transfection was performed with 50 nM of Silencer Select Predesigned trp53 (Ambion, siRNA ID s75472, Waltham, MA, USA), and Silencer Select Negative Control No. 1 siRNA (Ambion).

### RNA extraction, reverse transcription, and qPCR analysis

Total RNA was isolated using the RNeasy Mini Kit (Qiagen) according to the protocol provided by the manufacturer. One microgram of RNA was subsequently used for reverse transcription using the SensiFAST cDNA Synthesis Kit (Meridian Bioscience, BIO-65054), following the manufacturer’s guidelines. Quantitative real-time PCR (qRT-PCR) was conducted with Fast SYBR Green PCR Master Mix (Applied Biosystems). Relative gene expression levels were calculated using the 2^−ΔΔCt^ method, with normalization to the expression of mouse TATA-binding protein (TBP).

### Lipidomics

For lipidomics analysis via mass spectrometry (MS), KPC270 and KPC172 were transfected with siRNA against p53 or siRNA Negative Control. Cells were collected 72 h after trasnfection, and pellets containing 1 × 10^6^ cells per replicate were snap-frozen and stored at − 80 °C. Five biological replicates were prepared for each condition. Ultra-high-pressure liquid chromatography coupled to high-resolution tandem mass spectrometry (UHPLC-MS/MS—Vanquish and QExactive, Thermo Fisher, San Jose, CA, USA) was used for lipidomic analysis, as extensively described in prior technical notes [40].

## Data Availability

No datasets were generated or analysed during the current study.
